# Behavior Change Techniques Present in Wearable Activity Trackers: A Critical Analysis

**DOI:** 10.2196/mhealth.4461

**Published:** 2016-04-27

**Authors:** Kathryn Mercer, Melissa Li, Lora Giangregorio, Catherine Burns, Kelly Grindrod

**Affiliations:** ^1^ School of Pharmacy University of Waterloo Waterloo, ON Canada; ^2^ Kinesiology University of Waterloo Waterloo, ON Canada; ^3^ Faculty of Engineering University of Waterloo Waterloo, ON Canada

**Keywords:** older adults, physical activity, wearables, mobile health, chronic disease management

## Abstract

**Background:**

Wearable activity trackers are promising as interventions that offer guidance and support for increasing physical activity and health-focused tracking. Most adults do not meet their recommended daily activity guidelines, and wearable fitness trackers are increasingly cited as having great potential to improve the physical activity levels of adults.

**Objective:**

The objective of this study was to use the Coventry, Aberdeen, and London-Refined (CALO-RE) taxonomy to examine if the design of wearable activity trackers incorporates behavior change techniques (BCTs). A secondary objective was to critically analyze whether the BCTs present relate to known drivers of behavior change, such as self-efficacy, with the intention of extending applicability to older adults in addition to the overall population.

**Methods:**

Wearing each device for a period of 1 week, two independent raters used CALO-RE taxonomy to code the BCTs of the seven wearable activity trackers available in Canada as of March 2014. These included Fitbit Flex, Misfit Shine, Withings Pulse, Jawbone UP24, Spark Activity Tracker by SparkPeople, Nike+ FuelBand SE, and Polar Loop. We calculated interrater reliability using Cohen's kappa.

**Results:**

The average number of BCTs identified was 16.3/40. Withings Pulse had the highest number of BCTs and Misfit Shine had the lowest. Most techniques centered around self-monitoring and self-regulation, all of which have been associated with improved physical activity in older adults. Techniques related to planning and providing instructions were scarce.

**Conclusions:**

Overall, wearable activity trackers contain several BCTs that have been shown to increase physical activity in older adults. Although more research and development must be done to fully understand the potential of wearables as health interventions, the current wearable trackers offer significant potential with regard to BCTs relevant to uptake by all populations, including older adults.

## Introduction

### Chronic Illness, Physical Activity, and Sedentary Behavior

Physical inactivity contributes to an estimated 3.2 million deaths each year [1]. As of 2010, almost one in four adults was receiving less than the recommended 150 minutes of moderate-intensity physical activity per week [2]. Physical inactivity is the fourth leading cause of mortality, behind only hypertension, tobacco use, and high blood glucose [3]. The prevalence of physical inactivity is increasing, and it has been identified as a major risk factor for breast and colon cancers, diabetes, and heart disease [3]. Increased exercise can reduce frailty, lower blood pressure, and lead to a longer independent life [4,5].

Sedentary behavior is an independent risk factor for chronic disease that is separate from physical inactivity. Sedentary behavior is defined as “any waking behavior characterized by an energy expenditure of < 1.5 metabolic equivalents while in a sitting or reclining posture” [6]. For example, children who watch more than 2 hours of television a day have poorer body composition, physical fitness, self-esteem, prosocial behavior, and academic achievement [7]. Sedentary behavior is also associated with metabolic syndrome and cardiovascular disease in adults independently of physical activity [8,9].

### Wearable Activity Trackers

Wearable activity trackers are an emerging solution for motivating people to improve their physical activity levels and reduce sedentary behavior. Wearable trackers are activity monitors that track daily movement through sensors and companion smartphone or computer applications. As of 2015, at least half of consumers have heard of wearable activity trackers such as Fitbit or Jawbone and one in three have plans to purchase one [10]. And although the predicted buyers for most products are young people who are already living a healthy lifestyle [9], participants aged more than 60 years also appear to be receptive to using wearable activity trackers and learn to use them quite easily [10]. The newer-generation wearable activity trackers are also fairly accurate when compared with research-grade devices, [10] but only when used by people who do not have the atypical gaits often seen in those who experienced stroke or traumatic brain injury or have Parkinson's disease [11-16].

### Behavior Change Techniques

One question that emerges is how well wearable activity trackers align with the evidence-based techniques that have been shown to increase physical activity levels. One approach to identifying the behavior change techniques (BCTs) present in new and emerging technologies is to use a taxonomy such as the Coventry, Aberdeen, and London-Refined (CALO-RE) taxonomy. First published in 2011, the CALO-RE taxonomy contains 40 techniques derived from behavior change theories. It was based on an earlier 26-item taxonomy developed in 2008 by Abraham and Michie [17,18] and was refined using systematic reviews of physical activity and healthy eating interventions. The CALO-RE taxonomy was designed to help developers of new interventions identify and apply evidence-based techniques [19]. In 2013, Michie et al expanded the CALO-RE taxonomy to Behavior Change Technique Taxonomy (BCTT), which contains 93 items sorted into a hierarchy and is intended for multiple behaviors and disciplines (eg, health, environment) [18,20,21]. However, unlike BCTT, CALO-RE was specifically designed for physical activity and healthy eating behaviors and continues to be widely used [19,22]. Furthermore, the CALO-RE taxonomy has been widely used to characterize physical activity interventions such as smartphone apps [21], health coaching [19], and interventions for preventing and managing obesity in children [23].

Wearable activity trackers are being targeted at users of mobile apps as a way to promote physical activity [9]. However, there are key differences between mobile apps and wearable activity trackers. Direito et al [24] found that the 40 top-rated physical activity and diet apps most commonly provided instruction, set graded tasks, and prompted self-monitoring. In a similar study of 167 physical activity apps, Conroy et al [21] also identified that it was common for apps to model or demonstrate target behaviors and provide feedback on performance. More recently, Lyons et al [25] used the BCTT to systematically analyze 13 wearable activity trackers and found that all trackers helped users to self-monitor behavior, obtain feedback on behavior, and add objects to the environment while also generally supporting users in goal setting and comparing their behavior with their goal. Lyons et al [25] also found that wearable activity monitors contain a wide range of techniques that are typically used in clinical behavioral interventions and that the trackers are a medium by which these interventions may be translated into widespread use. However, one aspect not addressed by the above studies is self-efficacy. In recent years, self-efficacy has emerged as a priority for improving physical activity in older adults. Self-efficacy is “the belief in one's capabilities to organize and execute the courses of action required to produce given attainments” [26]. Theoretically, people who have a high self-efficacy for physical activity should be more likely to initiate, increase, and maintain physical activity, even in the face of obstacles and setbacks [26]. A 2011 systematic review identified self-efficacy as one of the most consistent predictors of physical activity in adults of all ages [27]. A 2009 literature review of interventions for older adults also found that self-efficacy was one of the most intensely studied and constant predictors of physical activity maintenance [28]. Furthermore, a 2014 systematic review by French et al [20] concluded that self-regulation techniques such as goal setting, feedback, and social support are effective for younger adults, whereas older adults may benefit more from problem-solving, rewards for successful behavior, and modeling or demonstrating behavior. Therefore, the objective of this study was to use the CALO-RE taxonomy to examine whether the design of wearable activity trackers incorporates BCTs. A secondary objective was to critically analyze if the BCTs present relate to known drivers of behavior change in older adults, such as self-efficacy.

## Methods

**Figure 1:**
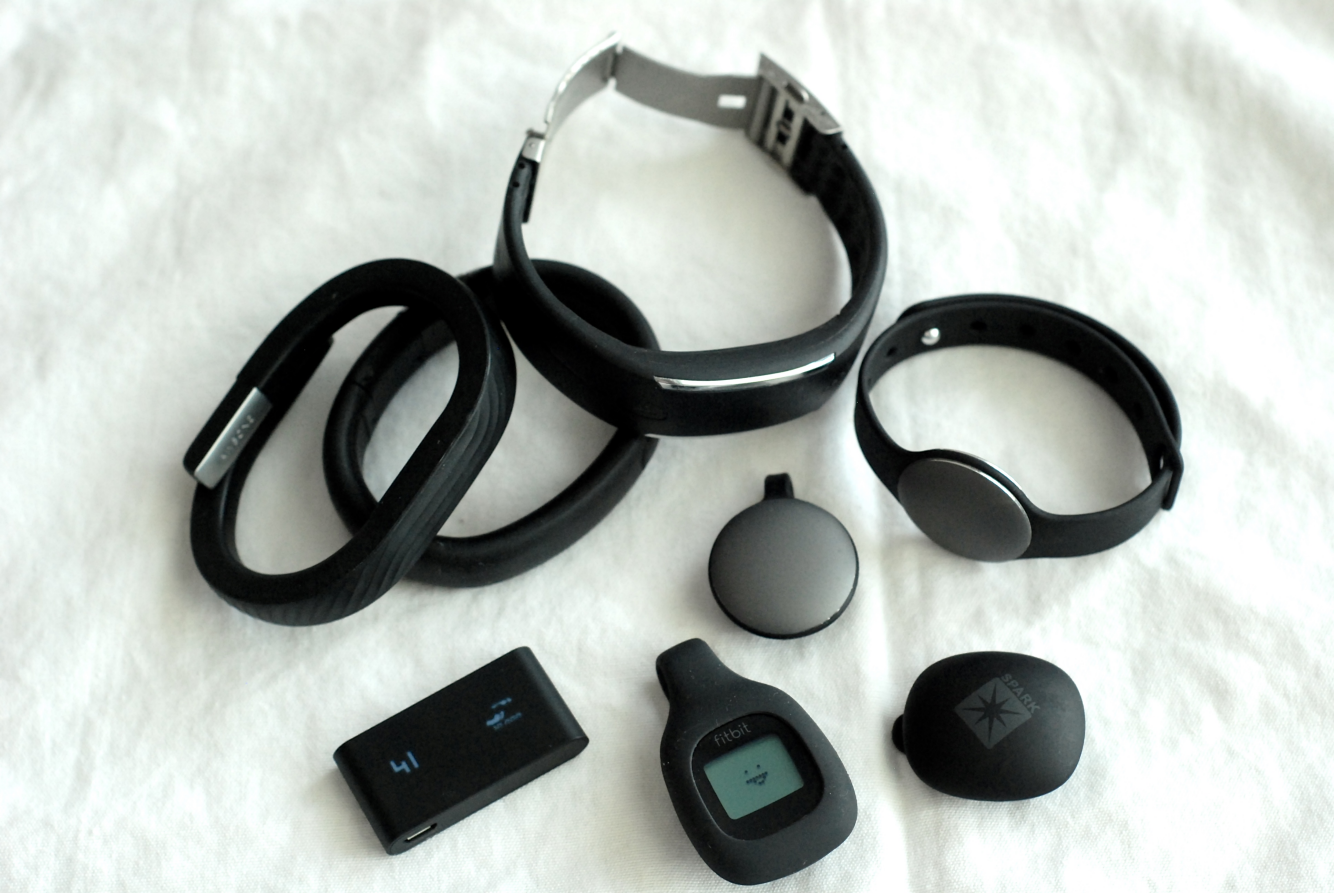
Wearable fitness trackers. Top L-R: Jawbone UP24, Nike Fuelband, Polar Loop, Misfit Shine with sport band . Centre: Misfit Shine with action Clip. Bottom L - R: Withings Pulse, Fitbit Zip, Spark Activity Tracker.

We ran this study concurrently with another study examining the real and perceived acceptance of wearable activity trackers by older adults (aged more than 50 years) living with chronic illness [29]. We wanted to analyze BCTs as they relate to the overall population, an added nod to the applicability to the older adult population. The team of researchers included a pharmacist (KG), a pharmacy student (ML), two systems design engineers (CB and LR), a kinesiologist (LG), and an information specialist (KM). We used the same trackers for both studies, as they reflected the available wearable fitness trackers in Canada. Our inclusion criteria for the wearable activity trackers included (1) continuous monitoring of some kind of physical activity outcome (steps, minutes of activity, points) and (2) provision of feedback via a separate mobile device or personal computer. We considered a device to be a wearable activity tracker if it contained an accelerometer and connected with a mobile platform. The device also had to be able to be wirelessly paired with handheld or desktop computers with at least Bluetooth 2.0 and be compatible with either Android 1.6+ or Apple's operating system iOS 6.0+. The following wearable activity trackers were evaluated for BCT content: Misfit Shine, Fitbit Flex, Jawbone UP24, Withings Pulse, Nike+ FuelBand SE, Polar Loop, and SparkPeople (Figure 1).

### Rating

We coded the wearable activity trackers using the CALO-RE taxonomy, which is a standardized tool for describing and comparing the BCTs used in lifestyle interventions [17]. As a test for calibration, the mobile apps Runkeeper and MyFitnessPal were coded using the CALO-RE taxonomy. To resolve differences in interpretation between raters, we discussed any ambiguous descriptors or definitions from the CALO-RE taxonomy until we reached agreement during the calibration process. This is correct For each wearable activity tracker, we downloaded the associated mobile app onto an Apple device (iPad mini, iPhone 5) and/or an Android device (Samsung Galaxy S4 smartphone or Google Nexus tablet). Two independent raters (ML and KG) wore each activity tracker for 1 week between May and August 2014 and rated the seven activity trackers and their associated mobile/Web-based platforms using the CALO-RE taxonomy. Images and website links that indicated the presence of a BCT were compiled during ratings and used later in discussion to resolve any conflicts between raters. If consensus was not reached after consulting the referenced item, a third user, who had used the activity tracker for at least 1 month, was brought in to resolve the disagreements. With 1 month of use, the third user was more likely to have been exposed to all possible taxonomy elements, if present, in the device.

We coded each technique using a dichotomous score of either 0 (not present) or 1 (present). For mobile apps and websites that had archives of information articles, we only coded a technique if the user was prompted to read a specific article or community post through an email alert or daily message. Upon installation of certain mobile apps, animations would prompt users to look at specific tabs and features or everything listed in the main menu. If any of these elements fit in with a BCT, it was also coded. We did not consider any information that was not immediately accessible or that was not presented through prompting.

### Statistical Analysis

We used descriptive statistics to summarize the CALO-RE ratings. We calculated interrater reliability using Cohen's kappa on SPSS Statistics (version 22, IBM). Cohen's kappa is used to describe the degree of interrater reliability between two raters and can be applied to dichotomous data [30]. We also calculated the total number of techniques present in each activity tracker and the frequency of each technique.

## Results

### Rating

**Figure 2:**
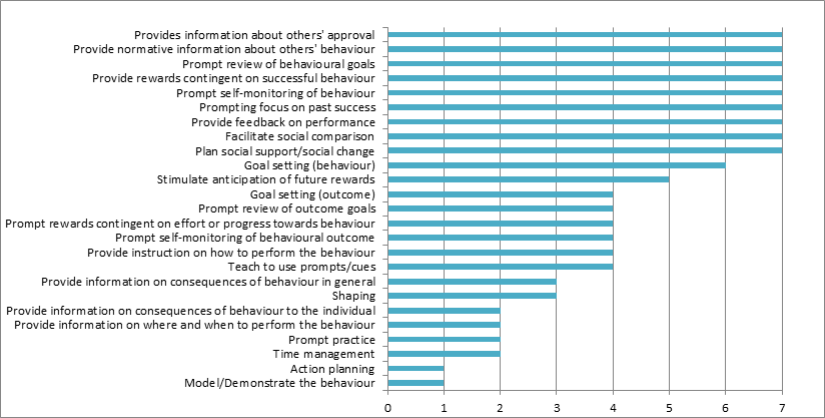
Most common behavior change techniques; with total number of devices found in.

Seven wearable activity trackers were rated by two independent raters using the 40-item CALO-RE taxonomy [18]. The number of BCTs ranged from 10 to 23, the most common shown in Figure 2. The 40 BCTs rated in each tracker were based on the CALO-RE taxonomy and included consequences of behavior, goal setting, problem-solving, outcome review, prompting, practicing, teaching, and social encouragement. As shown in Figure 2, a total of 9 techniques were present in every tracker. All trackers prompt users to self-monitor their activity levels, review goals, and review past successes while also providing feedback on performance, either by comparing step counts with user goals or by sending summary emails on activity levels over a certain period of time (daily, weekly, monthly). All trackers also included an optional social component to help users see others' approval or to plan for social support and change. Six trackers encouraged users to set a physical activity goal. Seven BCTs that were used less commonly included focusing on past and future performances, teaching prompts and cues, and instructing on how to perform a behavior.

There were 15 techniques absent in all wearable activity trackers, as listed in Textbox 1. The missing techniques were related to self-efficacy, planning, negative feelings (eg, fear), consequences, and comfort zones.

**Behavior change techniques absent in wearable activity trackers.**

Behavior change techniques absent

- Barrier identification or problem-solving
- Set graded tasks
- Prompting generalization of a target behavior
- Environmental restructuring
- Agree behavioral contract
- Use of follow-up prompts
- Prompt identification as role model or position advocate
- Prompt anticipated regret
- Fear arousal
- Prompt self-talk
- Prompt use of imagery
- Relapse prevention or coping planning
- Stress management or emotional control training
- Motivational interviewing
- General communication skills training

### Statistical Analysis

The kappa scores for interrater reliability of the taxonomy for the seven wearable activity trackers ranged from .746 to 1 (Table 1) [31]. According to the benchmarks assigned by Landis and Koch [30], the strength of agreement is “substantial” to “almost perfect.”

**Table 1:** Interrater reliability of taxonomy per wearable activity tracker

Wearable activity tracker	Cohen's kappa (95% CI)
Fitbit Flex	1 (0.00)
Jawbone UP24	.899 (0.764-1.034)
Misfit Shine	1 (0.00)
Nike+ FuelBand SE	.944 (0.836-1.052)
Polar Loop	.881 (0.722-1.040)
SparkPeopl Spark Activity Tracker	.746 (0.544-0.948)
Withings Pulse	.899 (0.764-1.034)

The mean number of BCTs incorporated in wearable activity trackers was 16.3/40 (SD 4.6), with Withings Pulse having the highest number at 23/40 and Misfit Shine having the lowest at 10/40 (Table 2). In case of Withings Pulse, most of the techniques, particularly those that focused on the provision of information, were addressed in a comprehensive “Frequently Asked Questions” section of the mobile app. All of the BCTs coded this way were related to the provision of information, such as “provide instruction on how to perform the behavior.” In comparison, for the SparkPeople device, which had 21/40 BCTs, most techniques were found in articles and videos available on the Web-based platform, which were brought to the attention of the user via email notifications. Misfit Shine and Polar Loop bands incorporated the lowest number of techniques, with only 10/40 and 13/40 techniques, respectively. This in part can be explained by the absence of outcome goals (eg, weight), minimal information in the accompanying mobile apps and websites, and a lack of a virtual reward system.

**Table 2:** Behavior change technique content of wearable activity trackers, mean 16.3 (SD 4.6).

Wearable activity tracker	Number of behavior change techniques (N=40)	Possible behavior change techniques present (%)
Withings Pulse	23	57.5
SparkPeople Spark Activity Tracker	21	52.5
Jawbone UP24	18	45.0
Fitbit Flex	15	37.5
Nike+ FuelBand SE	14	35.0
Polar Loop	13	32.5
Misfit Shine	10	25.0

## Discussion

Many of the newest generation wearable activity trackers include BCTs in the user interface. Results from the CALO-RE taxonomy ratings demonstrated that the devices tend to focus on techniques for goal setting, self-regulation, and social support. Techniques related to self-efficacy (such as planning, consequences, and knowledge) were present in less than half of the trackers or absent in all seven devices.

Our findings were similar to the ratings of activity trackers by Lyons et al [25]. However, there are some notable differences. For example, in our study, none of the reviewers identified problem-solving as a feature in the Jawbone tracker, whereas the coders in the study by Lyons et al did. Although Lyons et al [25] used the 93-item taxonomy, they recalculated their results with the CALO-RE taxonomy and found an average of 9 techniques across 13 activity trackers. In comparison, we found an average of 16 techniques, which suggests that identification of BCTs depends on how users experience the devices. While rating these devices, we were also actively testing the devices with individuals aged more than 50 years who were living with chronic illness. By concurrently using the devices alongside active users, we may have had the opportunity to identify more features than if we had limited our use of the trackers to the research team.

In previous evaluations of physical activity interventions among adults aged 50 years and older, social support from peer mentors, families, and friends significantly increased initiation and maintenance of physical activity [32]. Online communities have also been associated with increasing step counts in walking programs in study participants with an average age of 50 years and greater [33]. Behavior change techniques relating to social support were present in every wearable activity tracker and associated platforms through online communities and connectivity to popular social media networks.

A 2014 systematic review by French et al [20] identified that the three BCTs with the greatest effect on physical activity in older adults are problem-solving, rewards for successful behavior, and modeling or demonstrating the behavior. We found that rewards were present in all seven trackers, while behavior modeling was only present in one tracker, and problem-solving was not present in any of the trackers. French et al [20] also identified that interventions had the greatest effect on older adults when they provided a combination of normative information about others' behavior and information on where and when to perform behavior and helped participants plan social support or social change. We also found that two techniques (information about others' behavior and planning) were present in all seven trackers but that only two trackers helped users identify where and when to perform the behavior.

Self-efficacy is an important predictor for starting and maintaining physical activity in adults aged more than 50 years [28]. However, the effect of wearable activity trackers on self-efficacy is not clear, as O’Brien et al found no increase in physical activity self-efficacy in a 12-week study of older adults using Nike FuelBand [10]. The systematic review by French et al [20] found that the techniques associated with greater self-efficacy include prompt use of imagery, motivational interviewing, and prompt generalization of target behaviors, none of which were identified in the trackers in our study. This suggests that there is potential if self-efficacy techniques are increased in wearable activity trackers for increase in physical activity.

The focus of wearable activity trackers on self-monitoring and self-regulation is to be expected. The main design and purpose of these devices is to monitor past, present, and future activity. A qualitative analysis of an 18-month physical activity intervention in older adults found that targeting self-regulation behaviors may support long-term increases in physical activity [32]. All but one tracker required users to set an activity goal, often in the form of steps per day. Goal setting may also significantly increase physical activity among older adults, particularly if goals are specific and related to the desired behavior [20].

### Limitations

Although we had a high interrater reliability, the ratings may not represent the most current version of the wearable activity tracker and associated platforms because of frequent updates. We minimized these elements by ensuring that all notifications were enabled, saving screenshots and website links representing each technique, consulting an experienced tracker user when necessary, and downloading all updates as of August 8, 2014. This method of evaluating the physical tracker and downloaded platforms allowed us to identify more BCTs than if Web-based descriptions alone were used, which was the approach used by Conroy et al [21] to rate mobile apps for physical activity using the CALO-RE taxonomy.

One challenge for studies of this nature is that there is no guarantee that the user will encounter a technique even if it is present. For example, if a user does not wish to use or does not have access to social media, then the user will not encounter any of the tracker's social support techniques. A further challenge for rating trackers is that they are complex tools with multiple features and different users are likely to have different experiences. For example, a user who wants to perform more physical activity may not use the device in the same way as a user who wants to be less sedentary. Similarly, a user who has a lower health or technology literacy may not explore the features as deeply as a health professional or expert technology user, regardless of age. As a result, this study should be considered a snapshot of the BCTs of wearable activity trackers and of the potential of how these trackers can relate to self-efficacy in the older user. By determining the similarities and differences between the overall population and the older adult population, there is great potential to develop wearable activity trackers and their affiliated apps to be the most broadly reaching.

### Conclusions

Wearable activity trackers are a promising innovation for promoting physical activity behaviors in a wide age range of users, including the older adult population. They can easily be distributed across a wide population and integrated as a part of physical activity interventions through pharmacies and prescribers. Behavior change techniques most commonly found in the evaluated wearable activity trackers, such as self-monitoring and self-regulation techniques, are likely to appeal more to younger and middle-aged adults. To make wearable activity trackers more appealing to older adults, additional BCTs that are specific to older adult needs may be necessary, such as helping users find ways to overcome barriers to physical activity by problem-solving and modeling or demonstrating ways to increase physical activity. Future collaborations between tracker developers and health behavior change experts may enhance the potential to elicit behavior change.

## Appendix

Multimedia Appendix 1 CALO-RE Taxonomy Rating Data.

## Abbreviations

BCT: behavior change technique
BCTT: Behavior Change Technique Taxonomy
CALO-RE: Coventry, Aberdeen, and London-Refined
