# Supplementary material for: Behavior Change Techniques Present in Wearable Activity Trackers: A Critical Analysis
Source: JMIR Mhealth Uhealth. 2016 Apr 27;4(2):e40. doi: 10.2196/mhealth.4461 (PMC4917727; doi:10.2196/mhealth.4461)
Supplement: Multimedia Appendix 1 [file mhealth_v4i2e40_app1.pdf]

Appendix 1: CALO-RE Taxonomy Rating Data

	Wearable Activity Tracker						
Taxonomy Item	Fitbit Flex	Jawbone UP24	Misfit Shine	Nike+ Fuelband SE	Polar Loop	SparkPeople Activity Tracker	Withings Pulse
1	0	1	0	0	0	1	1
2	0	1	0	0	0	1	0
3	1	1	1	1	1	1	1
4	1	1	1	1	1	1	1
5	1	1	1	1	0	1	1
6	1	1	0	0	0	1	1
7	0	0	0	0	0	0	1
8	0	0	0	0	0	0	0
9	0	0	0	0	0	0	0
10	1	1	1	1	1	1	1
11	1	1	0	0	0	1	1
12	0	0	0	1	1	1	1
13	1	1	1	1	1	1	1
14	1	0	0	1	0	0	1
15	0	0	0	0	0	0	0
16	1	1	1	1	1	1	1
17	1	1	0	0	0	1	1
18	1	1	1	1	1	1	1
19	1	1	1	1	1	1	1
20	0	0	0	0	0	1	1
21	0	1	0	0	1	1	1
22	0	0	0	0	0	1	0
23	0	1	0	1	1	0	1
24	0	0	0	0	0	0	0
25	0	0	0	0	0	0	0
26	0	1	0	0	0	0	1
27	0	0	0	0	0	0	0
28	1	1	1	1	1	1	1
29	1	1	1	1	1	1	1
30	0	0	0	0	0	0	0
31	0	0	0	0	0	0	0
32	0	0	0	0	0	0	0
33	0	0	0	0	0	0	0
34	0	0	0	0	0	0	0
35	0	0	0	0	0	0	0
36	0	0	0	0	0	0	0

37	0	0	0	0	0	0	0
38	0	0	0	0	0	1	1
39	0	0	0	0	0	0	0
40	1	0	0	1	1	1	1
Total	**15**	**18**	**10**	**14**	**13**	**21**	**23**
